# Molecular Characterization of Norovirus Strains Isolated from Older Children and Adults in Impoverished Communities of Vhembe District, South Africa

**DOI:** 10.1155/2020/8436951

**Published:** 2020-06-29

**Authors:** G. Mulondo, R. Khumela, J. P. Kabue, A. N. Traore, N. Potgieter

**Affiliations:** ^1^Department of Microbiology, School of Mathematical and Natural Sciences, University of Venda, Thohoyandou, South Africa; ^2^School of Mathematics and Natural Sciences, University of Venda, Thohoyandou, South Africa

## Abstract

**Background:**

Human norovirus (NoV) is an etiological agent associated with acute gastroenteritis (AGE) in both children and adults worldwide. However, very few studies have been reported on the prevalence and genetic diversity of NoV strains in children older than 5 years of age and adults with little or inadequate water and sanitation conditions.

**Objectives:**

The aim of this study was assessing the prevalence of the human norovirus in older children and adults suffering with diarrhoea from rural communities in the Vhembe district, Limpopo province.

**Methods:**

Between August 2017 and October 2018, stool samples were collected from outpatients suffering from AGE and screened for NoV strains using the RIDA©GENE norovirus I and II real-time one-step RT-PCR. RNA extracts of NoV-positive samples were subjected to RT-PCR amplification and nucleotide sequencing to genotype the positive NoV strains.

**Results:**

Out of 80 collected stool samples, 13 (16%) were tested positive for norovirus. Genogroup GII was identified in 6/13 (46%) samples and genogroup GI in 7/13 (54%) samples. The sequence analyses showed multiple genotypes including GII.Pg, GII.1, GII.2, GII.4, and GI.3. Phylogenetic analysis revealed the relatedness of NoV genotypes identified with other strains reported globally.

**Conclusion:**

Continued systematic surveillance to evaluate norovirus association with diarrhoea is needed to assist with epidemiological surveillance and disease burden in people of all the age groups.

## 1. Background

Currently, norovirus (NoV) is recognized as the major leading cause of acute gastroenteritis in people of all the age groups [1, 2]. Several studies are available on the prevalence and distribution of NoV in developing countries. However, only few studies have been conducted in low-income countries [2–4]. There are also no data available on NoV infection in outpatients such as older children and adults from rural communities of South Africa, where most people live in poor environmental conditions with poor sanitation, lack of safe drinking water, and poor hygiene practices [2].

Norovirus is a small (approximately 38 nm in diameter), nonenveloped, positive-sense, single-stranded RNA (ssRNA+) genome of approximately 7.5 Kbs in length [5–7]. Based on the capsid protein VP1, norovirus is subdivided into ten genogroups (GI–GX) which include 49 genotypes, 2 tentative new genogroups (GNA1 and GNA2), and 3 tentative new genotypes (GII.NA1, GIINA2, and GIV.NA1) [8]. Based on the RdRp region of ORF1, norovirus is now subdivided into 60 P-types, 2 tentative P-groups, and 14 tentative P-types [8]. Norovirus from genogroups I, II, and IV is known to infect humans [9–12].

Norovirus is a causative agent of high mobility and morbidity rate in people of all the age groups [4, 13], and it has been estimated to be responsible for about 18% of norovirus-associated gastroenteritis outbreaks [14, 15] with approximately 212,000 death cases. The prevalence and distribution of norovirus have been conducted in different settings such as healthcare centres, hospitals, and communities. Norovirus was shown to account for 17% in inpatients than outpatients with 20% followed by community setting with 24% [15, 16]. In addition, NoV has shown to account for 6–27% of acute gastroenteritis in adults of different age groups in several studies conducted in Canada, China, the Netherlands, Portugal, Spain, Qatar, the USA, and the UK [15].

In African countries, Shioda and colleagues [17] provided data from a rural community of Kenya that showed NoV detection rates of 26.3%, 22.5%, 25.5%, 27.1%, and 16.7% in outpatients of 5–9 years, 10–17 years, 18–34 years, 35–49 years, and ≥50 years, respectively. In Egypt, from March 2006 to February 2007, NoV accounted for 17% (4/23) in patients between 5 and 9 years, 4% (1/28) in patients aged between 10 and 14 years, and 10% (2/21) in patients of ≥15 years of age [18]. As such, the majority of NoV studies is focused on children below the age of 5 years [3]. The objective of this study was therefore to determine the prevalence and genetic diversity of NoV in children older than 5 years of age as well as adults in rural communities of Vhembe District, South Africa, between 2017 and 2018.

## 2. Methods

### 2.1. Compliance with Ethical Standards

Approval for this study was obtained from the Ethics Committees of the Department of Health in the Limpopo province (Ref. 4/2/2) and University of Venda (Ref. SMNS/18/MBY/03). Written informed consent was obtained from each participant before stool collection. An interview by health professional nurses was conducted with the child's parents and the adult patients to gather information on personal details regarding the date of birth, gender, starting date of diarrhoea, and symptoms associated with patient illness such as abdominal pain cramps (APC), fever, and dehydration. This survey was a cross-sectional study.

### 2.2. Sample Collection

Samples were collected between August 2017 and October 2018 from Primary Health Care (PHC) clinics serving rural communities of the Vhembe district in Limpopo province, South Africa. Stool samples were randomly collected from symptomatic cases of diarrhoea in patients above 5 years attending PHC clinics. Sterile specimen containers were used for collection of the stool sample and kept at 4°C during the transportation to the University of Venda molecular laboratory and immediately refrigerated at −20°C prior to RNA extraction. The consistency of the stool according to the Bristol stool chart was documented [19]. Diarrhoea in this study was referred to as the passage of three or more loose or watery liquid stools within the preceding 24 hours [20]. Bloody stool diarrheal samples were excluded from this study.

### 2.3. Viral RNA Extraction and Norovirus Detection

Before RNA extraction, sample processing was done on each raw specimen by diluting the stool 1 : 10 in phosphate buffer saline (PBS, 0.01 M, pH 7.2) (Thermo Fisher Scientific, Waltham, Massachusetts, United States) and thoroughly vortexed having to allow proper mixing. The published Boom extraction method was used [21] to extract viral RNA from the faecal suspension. RNA extracts were stored at −20°C prior to NoV detection. Specific primers set for NoV GII and GI and PCR conditions as previously described [22] were performed for NoV detection using RIDA©GENE norovirus I and II real-time RT-PCR (R-Biopharm AG, Darmstadt, Germany). The kit provides an internal control to assess the RNA extraction efficiency and the PCR inhibition. The manufacturer does not provide the description of primers and probes which are aimed to amplify the ORF1-ORF2 junction area of norovirus [23]. RIDAGENE norovirus I and II multiplex real-time RT-PCR is a one-step real-time RT-PCR format where reverse transcription is followed by the PCR in the same tube. The kit has a limit of detection ≥50 RNA copies per reaction. In this study, a sample was considered positive if the Ct value obtained is less than 45. The RIDAGENE norovirus I and II has a high sensitivity and specificity for detection of norovirus in stool specimens from patients with diarrohea [24].

### 2.4. Genomic Amplification

Extracts which tested positive for norovirus by one-step real-time PCR were then subjected to RT-PCR amplification for the purpose of nucleotide sequencing. The specific oligonucleotide primer pairs G1SKF/G1SKR to amplify 330 bp of the capsid region of NoV GI and G2SKF/G2SKR to amplify 344 bp of the capsid region of NoV GII were used to perform One-Step Ahead RT-PCR (QIAGEN, GmbH, Germany) as previously described [22, 25]. In addition, 326 bp of the RdRp fragment was amplified using primer set JV12/JV13 with the same PCR conditions as of G1SK primers. Designed primers (WGS 9F/WGS 9R) were used to amplify a 751 bp product of the GII capsid that were not detected by G2SK primers. Amplification conditions published previously were used [25], and all PCR products were analysed using a 2% (w/v) agarose gel in TAE buffer (40 mM Tris acetate; 20 mM acetic acid, 1 mM EDTA, and pH 8.3) stained with ethidium bromide. The resulted PCR products were purified with a master mix of ExoSAP (Nucleics, Australia).

### 2.5. Sequence Analysis and Phylogenetic Analysis

Sanger sequencing was performed on ABI 3500XL Genetic Analyzer POP7™ (Thermo Scientific) using the same specific primers described previously [22]. The raw sequence reads were edited with Finch TV v1.4 (Geospiza, Seattle, USA). The nucleotide sequences obtained from the selected NoV strains were used to search similar sequences in the NCBI genetic database using the BLAST tool (available at http://www.ncbi.nlm.nih.gov/) and then aligned using Noronet typing tools [26] (available at http://www.rivm.nlm/norovirus/typingtool). The reference strains from GenBank were randomly selected among the BLAST hits with >80% similarities on the query sequence of the NoV strains identified from this study. Phylogenetic trees were constructed by the neighbour-joining method [27] using MEGA 7 software, with 1,000 bootstrap replicates for each gene [28]. The evolutionary distances were computed using the *P*-distance method [29].

### 2.6. Statistical Analyses

Data were captured in Microsoft Excel. The *t*-test to compare cycle threshold (CT) values in diarrheal cases was performed using Paleontological Statistics (PAST) version 3.0. A *p* value <0.05 was statistically significant.

## 3. Results

### 3.1. Study Characteristics

A total of 80 stool specimens were randomly collected at different clinics in the rural communities of Vhembe District, Limpopo Province, South Africa. The age of patients ranged between 5 years and 68 years. More stool samples were collected from females than males. The major clinical symptoms associated with NoV infection in this study were diarrhoea with mixed symptoms (6/13; 46%) followed by diarrhoea with abdominal cramp pain (5/13; 38.5%) (Table 1). Mixed symptoms included vomiting, fever, and dehydration. Norovirus is mostly detected in watery stool samples (6/13; 46%).

### 3.2. Prevalence and Characterisation of Norovirus Strains

Out of 80 diarrheal faecal samples, 13 cases were tested positive for NoV (16%) (Table 1). Norovirus was mostly detected in adult females. Norovirus GII was detected in 6 (46%) samples, and norovirus GI was detected in 7 (54%) samples. The internal control recovery rate was 33.25 ± 0.97 (mean ± standard deviation (sd)). A significant difference (Student's *t*-test, unpaired, *t* = −2.60, *p* < 0.001) was observed between the CT values of GII genotypes and GI genotypes with their mean of 22.19 (*n* = 6, sd = 5.59) and 32.23 (*n* = 7, sd = 7.78), respectively.

Four (4) of the 13 positive NoV samples were successfully sequenced. Based on the capsid sequences and the polymerase region of NoV GII, 5 genotypes were identified for GII (Table 2), consisting of

- GII.1, GII.2, and GII.4 genotypes for the sequence with only the capsid fragment available;
- GII.Pg for the sequences with only the RdRp fragment gene available; and
- GII.Pg/GII.1 which is a putative recombinant for the sequence with both the polymerase and the capsid fragment. However, sequencing of the full junction region of ORF1/ORF2 is required to confirm the recombination.

One genotype was identified for GI, namely, the GI.3 strain, with only the capsid fragment identified (Table 2).

### 3.3. Phylogenetic Analyses of NoV Strains

**Figure 1:**
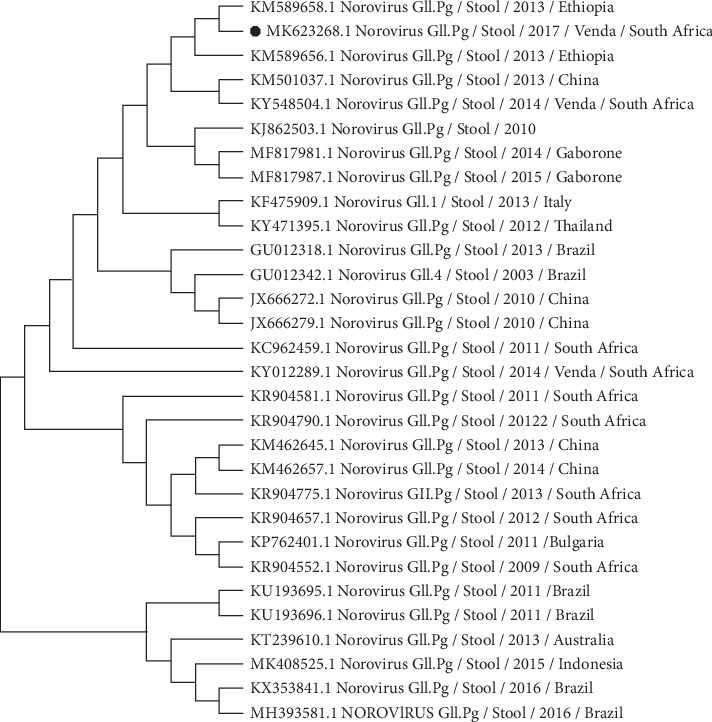
Phylogenetic tree based on the 326-nucleotide sequence of the NoV GII polymerase gene fragment; the neighbour-joining tree of the GII polymerase genotypes circulating between August 2017 and October 2018 in the rural communities of Vhembe District, Limpopo Province, South Africa. Round black dot indicates the GII.Pg polymerase genotype from this study. Thirty reference strains of NoV are randomly selected from GenBank with their respective accession numbers. All positions containing gaps and missing data are eliminated. Evolutionary analyses are conducted in MEGA X [30].

**Figure 2:**
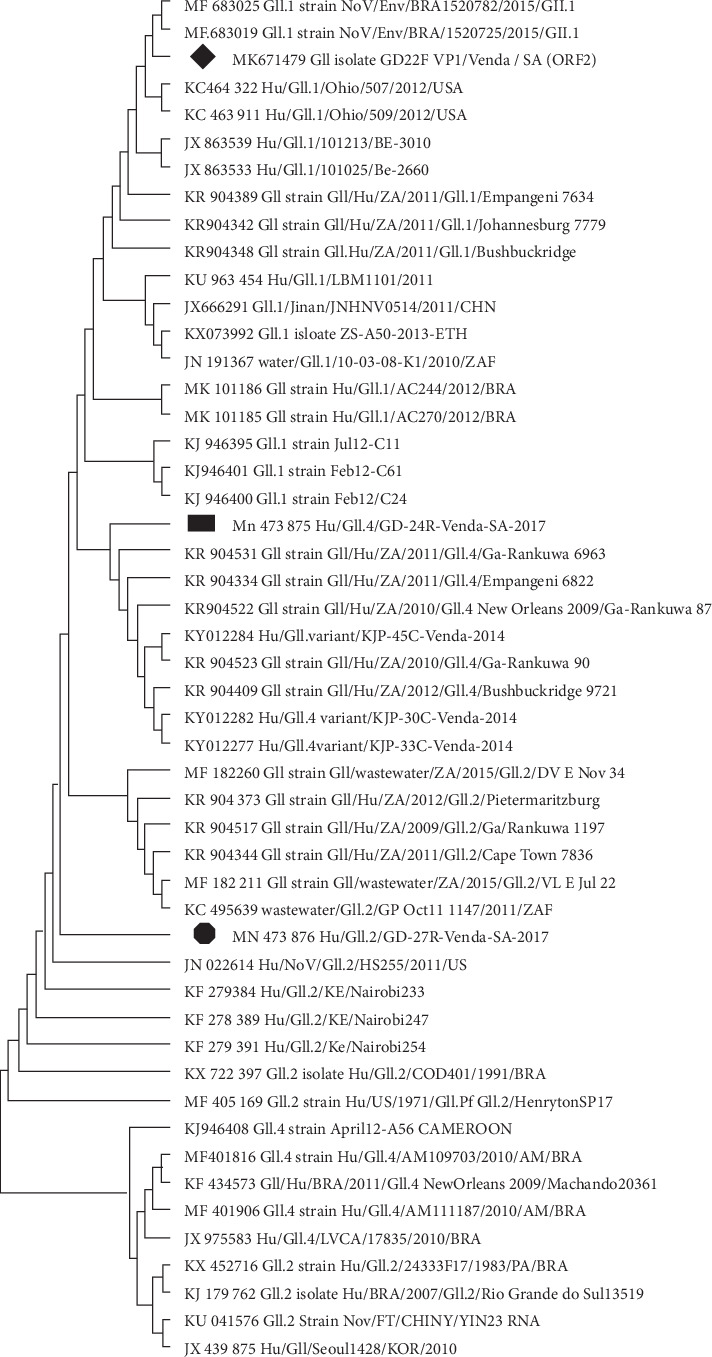
Phylogenetic tree based on the 344-nucleotide sequence of the NoV GII capsid gene fragment; the neighbour-joining tree of the GII capsid strains circulating between August 2017 and October 2018 in the rural communities of Vhembe District, Limpopo Province, South Africa. Squared black dot indicates the GII.4 capsid genotype, round black dot for the GII.2 genotype, and black diamond dot for the GII.1 capsid genotype. Forty-seven reference strains of NoV are selected from GenBank with their respective accession numbers. Evolutionary analyses are conducted in MEGA X [30].

**Figure 3:**
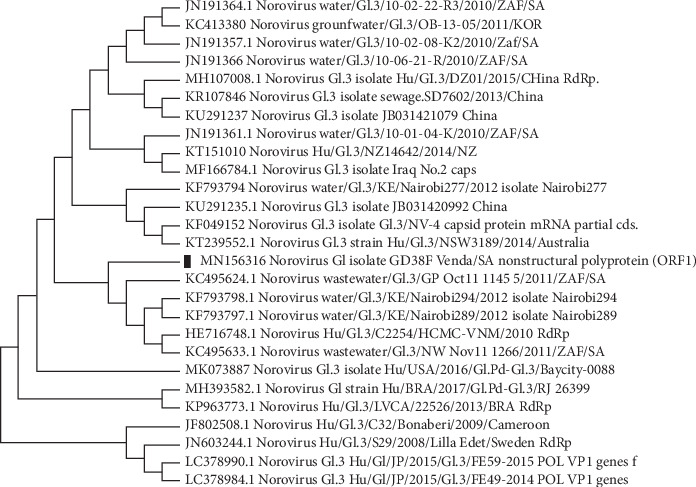
Phylogenetic tree based on the 330-nucleotide sequence of the NoV GI capsid gene fragment; the neighbour-joining tree of the GII capsid strains circulating between August 2017 and October 2018 in the rural communities of Vhembe District, Limpopo Province, South Africa. Squared black dot indicates the GI.1 capsid genotype. Twenty-seven reference strains of NoV are selected from GenBank with their respective accession numbers. Evolutionary analyses are conducted in MEGA X [30].

The similarity of all the reference strains used for the phylogenetic analyses in this study was ranging from 89 to 100% (Figures 1–3). Though with the limited number of sequences from our sequencing set, the results of this study reveal a genetic diversity of NoV in diarrheal stool samples collected from patients living in rural communities of the Vhembe district. Nucleotide sequences of the capsid and polymerase region of five GII and one GI NoV strains were submitted to GenBank. GenBank accession numbers for the nucleotide sequences are as follows: MK623268; MK671479; MN156316; MN473875; and MN473876.

## 4. Discussion

For the development of the successful NoV vaccine, identification of the target age groups that are more prone to NoV infection should be investigated, and knowledge on the circulating genotype of NoV in people of different age groups should also be known [31–33].

In this study, norovirus was detected amongst people of different age groups. Most cases were detected in older children. In adults with ≥19 years of age, NoV was detected only among the females (Table 1). NoV in adult women may be because younger people such as infants and children are more susceptible to NoV infections because they are more exposed to the contaminated environment, and they have not acquired enough immunity. Thus, it is likely that NoV infection spreads among young people to adults and elderly more especially females who are childminders. The major clinical symptoms associated with NoV infection in this study were diarrhoea with abdominal cramp pain followed by diarrhoea with mixed symptoms (Table 1). Mixed symptoms included vomiting, fever, and dehydration. Norovirus was mostly detected in watery stool. This suggests high NoV detection in liquid stool as previously reported in the study area [22].

Different genotypes of NoV were identified including GII.Pg, GII.Pg/GII.1, GII.1, GII.2, GII.4, and GI.3 in this study:

- GII.Pg NoV has been circulating before 2008 following an outbreak which occurred in Victoria in 1983 [34]. GII.Pg genotype is associated with outbreaks in both healthcare and nonhealthcare settings worldwide [34]. Reports of the detection of GII.Pg norovirus in humans as previously described [34] include studies in Australia [23, 35], Belgium [36], Spain [37], Taiwan [38], Germany [39], Italy [40], China [41], France [42], Tanzania [43], and South Africa [44]. The phylogenetic analysis revealed that the GII.Pg strain shares common ancestors with other common strains circulating in Africa including Ethiopia and Gaborone (Figure 1). Population movement within the African continent may facilitate the circulation of NoV strains on the continent.
- In the current study, a putative NoV recombinant genotype (GII.Pg/GII.1) was identified in 1 out of 5 (20%) of the successfully sequenced amplicons. The recombinant form of GII.Pg/GII.1 had been previously reported in South Africa [2] and other countries such as France [42], Germany [39], Indonesia [45], and Thailand [46]. This is an open door to the genetic variation of NoV strains. In Brazil, the GII.Pg/GII.1 putative recombinant strain was suggested to cause more severe symptoms [47]. Therefore, combined characterization of both capsid and polymerase regions is substantial to monitor the new recombinant strains and the new emerging NoV genotype worldwide.
- GII.1 capsid genotype found in this study has been recently reported in Cameroon [48], Brazil [49, 50], Australia [51], India [52], and elsewhere [53–55]. This genotype is frequently reported from both clinical cases and environmental samples. In the phylogenetic tree, GII.1 clustered together with the genotypes from environmental samples (Figure 2). This could be supported by Motoya et al.'s [56] speculation that both GI and GII strains can be detected in environmental samples.
- GII.2 genotype reported in this study has been previously reported more common in hospitalised older children than in younger children [31, 57, 58]. Between 2016 and 2017, the GII.2 genotype was predominant in China [14] and Cameroon [48]. This study finding is in agreement with the previous study done by Bruggink et al. [31] who reported GII.2 as the common variant in children suffering from AGE. Also, the GII.2 strain identified in this study was closely related with other GII.2 genotypes reported in other African countries (Figure 2).
- NoV GII.4 strains have been previously reported as the predominant genotype associated with sporadic cases or occasional outbreaks [59, 60]. Identified GII.4 genotype detected in this study is closely related to the South Africa/2010–2016 strains (Figure 2). Phylogenetic analysis of the GII.4 strain from our study and those which have been previously reported shows the continuous circulation of the GII.4 variant among the rural communities in the northern part of South Africa.
- Norovirus GI strains have been previously associated with NoV-related waterborne outbreaks [61]. Esteves et al. [62] reported NoV GI.3 as the most frequently detected strain in Africa after the predominantly circulating NoV GII.4 strain. In the present study, GI.3 was detected only in one sample and was found to be closely related to other strains reported in South Africa (Figure 3). As mentioned above, norovirus GI strains are mostly associated with environmental samples than clinical samples. Though the samples were from clinical specimens, the NoV GI.3 genotype in this study could be due to an exposure of contaminated environmental water.

One of the limitations is the small number of diarrheal cases collected. This is mostly due to the reluctance of adults to provide stool specimens for analysis. Only stool samples from patients with AGE were evaluated in this study. Therefore, we do not know the occurrence of NoV in healthy or asymptomatic individuals.

## 5. Conclusion

To the best of our knowledge, this is the first study to present the data on NoV prevalence and diversity in older children and adult patients living in rural communities of the Vhembe district. The study shows the circulation of different human NoV genotypes in patients older than 5 years of age, suggesting that preventive measures should be taken against norovirus infection in older patients.

## Figures and Tables

**Table 1:** Demographic profile (age and sex distribution) of norovirus strains detected in older children and adult patients living in rural communities of the Vhembe district, Limpopo province.

Age of patients (years)	No of stool samples collected (%)	Sex of patients		No of stool samples positive for NoV (%)	Sex of positive NoV samples (%)		Clinical symptoms in positive samples				Stool type of positive samples		
		Male	Female		Male	Female	Diarrhoea only	Diarrhoea and vomiting	Diarrhoea and APC^*∗*^	Diarrhoea and other symptoms	Watery	Formed	Soft
5–10	17/80 (21%)	13	4	5/17 (29%)	3	2	0	0	1	4	1	2	2
11–18	16/80 (20%)	9	7	1/16 (6%)	0	1	1	0	0	0	1	0	0
19–21	3/80 (4%)	1	2	0/3 (0%)	0	0	0	0	0	0	0	0	0
22–30	7/80 (9%)	2	5	0/7 (0%)	0	0	0	0	0	0	0	0	0
31–40	14/80 (18%)	3	11	1/14 (7%)	0	1	0	0	0	1	0	0	1
41–50	11/80 (14%)	3	8	2/11 (18%)	0	2	0	0	2	0	2	0	0
≥50	12/80 (15%)	1	11	4/12 (33%)	0	4	1	0	2	1	2	0	2
**Total**	**80/80 (100%)**	**32**	**48**	**13/80 (16%)**	**3**	**10**	**2**	**0**	**5**	**6**	**6**	**2**	**5**

^*∗*^APC, abdominal pain cramp; NoV, norovirus.

**Table 2:** Genotype distribution of the identified NoV strains in stool specimens between September 2017 and October 2018 in rural communities of the Vhembe district, South Africa.

Genogroup	Genotypes			Total
	RdRp	Capsid	RdRp/capsid	
GI		GI.3 (100%)		1 (100%)

GII	GII.Pg (100%)	GII.1 (33.3%)	GII.Pg/GII.1 (100%)	5 (100%)
		GII.2 (33.3%)		
		GII.4 (33.3%)		

## Data Availability

All nucleotide sequences of capsid and polymerase regions of five GII and one GI NoV strains were submitted to GenBank with assigned accession numbers as follows: MK623268; MK671479; MN156316; MN473875; and MN473876. The questionnaire data as well as the stool consistency data used to support the findings of this study are included within the article.
